# Chemical composition, anthelmintic, antibacterial and antioxidant effects of *Thymus bovei* essential oil

**DOI:** 10.1186/s12906-016-1408-2

**Published:** 2016-10-26

**Authors:** Nidal Jaradat, Lina Adwan, Shadi K’aibni, Naser Shraim, Abdel Naser Zaid

**Affiliations:** 1 Department of Pharmacy, Faculty of Medicine and Health Sciences, An-Najah National University, Nablus, State of Palestine P.O. Box 7,; 2 College of Pharmacy Nursing and Health Professions, Birzeit University, Birzeit, State of Palestine P.O. Box 14,; 3 Center of Birzeit University Testing Laboratories, Birzeit University, Birzeit, State of Palestine P.O. Box 14,

**Keywords:** *Thymus bovei*, Essential oil, Antioxidant, Anthelmintic, Antimicrobial

## Abstract

**Background:**

It has been recently recognized that oxidative stress, helminth and microbial infections are the cause of much illness found in the underdeveloped, developing and developed countries. The present study was undertaken to identify the chemical composition, and to assess anthelmintic, antimicrobial and antioxidant effects of *Thymus bovei* essential oil.

**Methods:**

The chemical composition of the essential oil was analyzed using gas chromatography mass spectrometry (GC-MS). Antimicrobial activity was tested against the selected strains from American Type Culture Collection (ATCC) and clinical isolates such as *Staphylococcus aureus*, *Escherichia coli, Pseudomonas aeruginosa,* Methicillin Resistant *Staphylococcus aureus*, *Candida albicans* using MIC assay. The anthelmintic assay was carried out on adult earthworm (*Pheretima posthuma*), while antioxidant activity was analyzed using the 2,2-diphenyl-1-picrylhydrazyl (DPPH) free radical scavenging method.

**Results:**

Trans-geraniol (35.38 %), α-citral (20.37 %) and β-citral (14.76 %) were the major compounds comprising 70.51 % of the essential oil. Our results showed that *T. bovei* essential oil exhibited strong anthelmintic activity, even higher than piperazine citrate, the used reference standard, with potential antioxidant activity almost equal to the Trolox standard. Furthermore, *T. bovei* essential oil had powerful antibacterial and antifungal activities against the studied pathogens.

**Conclusion:**

Essential oil of *T. bovei* exerted excellent antioxidant, antimicrobial, and anthelmintic activities. Moreover, this study found that *T. bovei* volatile oil contains active substances that could potentially be used as natural preservatives in food and pharmaceutical industries, these substances could also be employed for developing new anthelmintic, antimicrobial and antioxidant agents.

## Background

Essential oils are highly valuable natural products and most of them are phytogenic compounds and had been used since ancient times in cosmetics, food and medicine due to their aromatic odor as well as their therapeutic effect [1, 2].

Chemically, most of the essential oils are mixtures of hydrocarbon compounds (C_5_H_8_)_n_ with their oxygenated, hydrogenated and dehydrogenated derivatives and most of them are monoterpenoids and sesquiterpenoids. There are about 3000 species of plants containing essential oils, from which 10 % are commercially important and used in perfumes, cosmetics and medicines or used as food and drugs preservatives and flavoring agents [3–6].

Therapeutically, essential oils have been approved for their antibacterial, antifungal, antiviral, anthelmintic, local anaesthetic, antispasmodic, carminative, expectorant, antimutagenic and many other therapeutic effects [7, 8]*.*

It is well known that plant biodiversity is greatly affected by geographic location. The Holy Land, especially, West Bank-Palestine, is considered one of the richest areas in plant biodiversity. This may be due to its special climate and topo-geographic terrains. In fact, this location is covered with thousands of plant species. Since ancient time, Jericho was highly advanced in the field of agriculture and horticulture, where peas, lentils, barley and wheat were cultivated. For centuries, thyme was considered one of the most used herbs in the Palestinian folkloric food and medicine. The genus *Thymus* (Lamiaceae family) is known in several Mediterranean and other countries as food and a spice and is employed for the treatment of several diseases including cough, cancer and many fungal skin infections [9–11].

*Thymus bovei* Benth. is a native species of the eastern Mediterranean regions. It is an aromatic, perennial herbaceous plant and is considered one of the most important *Thymus* species for culinary and medicinal purposes. In the West Asian and North African regions, this herb has been utilized for treatment of various skin and blood infections as well as other infectious diseases. In the Palestinian folk medicine *T. bovei* has been used as anthelmintic, expectorant, antispasmodic, antiseptic as well as for treatment of injuries, upper respiratory system inflammations and anorexia [12].

To the best of our knowledge, there are no previous reports on the chemical composition and biological activity of *T. bovei* essential oil originating from this area. The aim of this study was to characterize the composition of the essential oil of wild-growing *T. bovei* from the city of Jericho in Palestine. The goal was also to test the anthelmintic, antioxidant, antifungal and antibacterial activities of the analyzed essential oil as a potentially new source of biologically active natural products, especially that this plant has been used in the Palestinian folk medicine from ancient times as anthelmintic, anti-inflammatory and as antiseptic.

## Methods

### Chemicals and reagents

The following reagents were used to evaluate the antioxidant activity: methanol (Lobachemie, India), n-hexane (Frutarom LTD, Haifa), Trolox ((s)-(−)-6 hydroxy-2,5,7,8-tetramethychroman-2-carboxylic acid) (Sigma-Aldrich, Denmark), and (DPPH) 2,2-Diphenyl-1-picrylhydrazyl (Sigma-Aldrich, Germany).

Reagents used for the screening of the antimicrobial activity of *T. bovei* volatile oil included nutrient broth that was ordered from Himedia, India, and Dimethyl sulfoxide (DMSO), which was purchased from Riedeldehan, Germany.

While ethanol (Lobachemie, India) and piperazine citrate (Sigma-Aldrich, Denmark) were the used reagents for the anthelmintic assay.

### Instrumentations

In this study, the following instruments were used: Gas Chromatography Mass Spectrometry (GC-MS) (QP-5000 GC-MS Shimadzu, Japan), ultrasonic-microwave cooperative extractor/reactor (CW-2000, China), rotary evaporator (Heidolph OB2000, VV2000, Germany), UV visible spectrophotometer (Jenway 7315, England), grinder (Moulinex model, Uno. China), balance (Radw ag, AS 220/c/2, Poland), filter paper (Machrery-Nagel, MN 617 and Whatman no.1, USA), micropipettes (Finnpipette, Finland), incubator (Nuve, Turkey), syringe filter 0.45 μm pore size (Microlab, China) and 96-well plates (Greiner bio-one, North America).

### Collecting and preparing plant materials

The aerial plant parts (leaves, stems and flowers) were collected during its flowering time from Jericho region (Palestine) in May, 2015. Botanical identification was carried out by Pharmacognosist Dr. Nidal Jaradat in the Pharmacognosy and Herbal Products Laboratory, Faculty of Medicine and Health Sciences, An-Najah National University, Palestine. The identification process was conducted using live herbal specimens and photographs from books. Voucher specimens were deposited in the Pharmacognosy and Herbal Products Laboratory under the code number: Pharm-PCT-2432.

To extract the volatile oil, the aerial parts of *T. bovei* were separated carefully and then washed twice using distilled water to avoid contamination. The washed parts were cut into small pieces for future use.

### Essential oil isolation

The microwave-ultrasonic method is a recently developed method used for the extraction of essential oils from medicinal plants. In this method, the volatile oil was extracted using a microwave oven with ultra-sonication. However, during the extraction process, the powder suspension being extracted was exposed to ultrasonic waves to improve the extraction process. In this study, the apparatus consisting of a microwave oven combined with an ultrasonic extractor was used (Ultrasonic-microwave Cooperative Extractor/Reactor (CW-2000, China)). A one L round bottom flask containing about 100 g of the plant was placed in this apparatus. In this flask, the plant was suspended in about 500 ml deionized water. Then, the flask was connected with Clevenger apparatus, which was placed in the same apparatus. While carrying out the extraction process, the power of the microwave-ultrasonic extractor apparatus was adjusted at 1000 W. The ultrasonic power of the apparatus was adjusted at its maximum power as well (50 W and at a frequency of 40 kHz). The extraction process using this apparatus was conducted for 10 min at 100 °C. This process was repeated three times. The obtained volatile oil was collected into a clean beaker, chemically dried and stored in well closed dark amber colored bottles in the refrigerator at −4 °C. The yield of this extraction method was 1.4 % w/w based on the fresh weight.

### GC-MS analysis

GC-MS chromatograms were recorded using Shimadzu QP-5000 GC-MS. The GC was equipped with Rtx-5 ms column (30 m long, 0.25 μm thickness and 0.250 mm inner diameter). Helium was used as a carrier gas at a flow rate of 1 ml/min. Injector temperature was 220 °C. Oven temperature was programmed from 50 °C (1 min hold) at 5 °C/min to 130 °C, then at 10 °C/min to 250 °C and kept isothermally for 15 min. Transfer line temperature was 290 °C. For GC-MS detection, an electron ionization system, with detector volts of 1.7 KV was used. A scan rate of 0.5 s, and scan speed 1000 amu/s was applied, covering a mass range from 38–450 M/Z.

### Identification of components

The chemical components of the essential oils were identified by comparing their MS to the reference spectra in the computer library (NIST) and also by comparing their retention indices and Kovats index in the literature. The quantitative data were obtained electronically from integrated peaks, area percentage without the use of correction factor [13].

### DPPH radical-scavenging activity

A stock solution of *T. bovei* essential oil was used to prepare stock solutions in methanol and Trolox, at a concentration of 1 mg/ml. Each of these stock solutions was diluted in methanol to prepare 12 working solutions with the following concentrations: 1, 2, 3, 5, 7, 10, 20, 30, 40, 50, 80, 100 μg/ml. A fresh solution of prepared DPPH (0.002%w/v) was mixed with methanol and with each of the above mentioned working solutions at 1:1:1 ratio. In addition, a negative control solution was prepared by mixing the above mentioned DPPH solution with methanol in 1:1 ratio. Then, all of these solutions were incubated at room temperature in a dark cabinet for 30 min. The optical density of these solutions was determined by using spectrophotometer at a wave length of 517 nm using methanol as the blank solution. The antioxidant activity of the essential oil and Trolox were determined using the following formula:

Percentage of inhibition of DPPH activity (%) = (A-B)/A × 100 %, where: A = optical density of the blank and B = optical density of the sample.

The antioxidant half-maximal inhibitory concentration (IC_50_) for the *T. bovei* essential oil and Trolox solutions as well as their standard deviations, were calculated using BioDataFit edition 1.02 (data fit for biologist) [14].

### Antimicrobial tests

The essential oil of *T. bovei* was investigated for its antibacterial and antifungal activities. The antibacterial activity was examined against the growth of four reference bacterial strains obtained from the American Type Culture Collection (ATCC); *Staphylococcus aureus* (ATCC 25923), *Escherichia coli* (ATCC 25922) and *Pseudomonas aeruginosa* (ATCC 27853) as well as against the growth of a diagnostically-confirmed Methicillin Resistant *Staphylococcus aureus* (MRSA) clinical isolates. The antifungal activity of *T. bovei* essential oil was examined against the growth of a diagnostically-confirmed *Candida albicans* clinical isolate. The antimicrobial activities of *T. bovei* oil used in this study were determined by using broth micro-dilution method as described previously by Wikler 2007 [15]. The essential oil of *T. bovei* was dissolved in 5 % Dimethyl sulfoxide (DMSO) at a concentration of 132 mg/ml. The prepared oil solution was filter-sterilized and then was serially micro-diluted (2 folds) 11 times in sterile nutrient broth. The dilution processes were carried out under aseptic conditions in 96 well plates. In the micro-wells that were assigned to evaluate the antibacterial activities of the oil, the concentration of this oil ranged from 0.129 to 66 mg/ml. While the concentrations of this oil in the micro-wells assigned to evaluate their antifungal activities ranged from 0.065 to 55 mg/ml. In these plates, micro-well number 11 contained essential oils-free nutrient broth, which was used as a positive control for microbial growth. On the other hand, micro-well number 12 contained essential oils-free nutrient broth that was left un-inoculated with any of the test microbes. This well was used as a negative control for microbial growth. Micro-wells numbers 1 to 11 were inoculated aseptically with the test microbes. At the time of inoculation, the final concentrations of microbial cells were about 5 × 10^5^ and 0.5–2.5 × 10^3^ colony-forming unit (CFU)/ml for the tested bacterial pathogens and *Candida albicans*, respectively. Each of the included microbes in this study was examined in duplicate for being inhibited by the obtained *T. bovei* essential oil.

All of the inoculated plates were incubated at 35 °C. The incubation period lasted for about 18 h for those plates inoculated with the test bacterial strains and for about 48 h for those plates inoculated with *Candida albicans*. The lowest concentration of *T. bovei* essential oil, at which no visible microbial growth in that micro-well was observed, and was considered as the minimal inhibitory concentration (MIC) of the examined *T. bovei* essential oil.

### Anthelmintic activity

Due to its physiological and anatomical resemblance to humans, intestinal roundworm parasites adult earthworms *Pheretima posthuma* (10 cm long) were used to evaluate the anthelmintic activity of *T. bovei* essential oil at different concentrations. Anthelmintic assay was carried out by dividing the earth worms into eight groups containing 10 worms in each group, five different concentrations of the essential oil (10, 40, 50, 75, and 100 mg/ml) in distilled water were prepared for the study. Distilled water was used as control. The reference drug piperazine citrate were also prepared in distilled water at 10 mg/ml. The time of paralysis was recorded when no movement was observed even after vigorous shaking. The time of death of worms was recorded after ascertaining that the worms neither moved when shaken vigorously nor when dipped in warm water (50 °C). The anthelmintic activity was determined by the method followed by Dash et al. 2002 [16].

### Statistical analysis

Essential oil yield, anthelmintic and IC_50_ values were determined in triplicates. Results were expressed as means ± standard deviation (SD). Data were analyzed using ANOVA with multiple comparisons. The statistical significance was determined when the *p* value was <0.05. Statistical significance was expressed using * with *p* value <0.05, ** with *p* value ≤ 0.001, and *** with *p* value ≤ 0.0001.

## Results

**Fig. 1:**
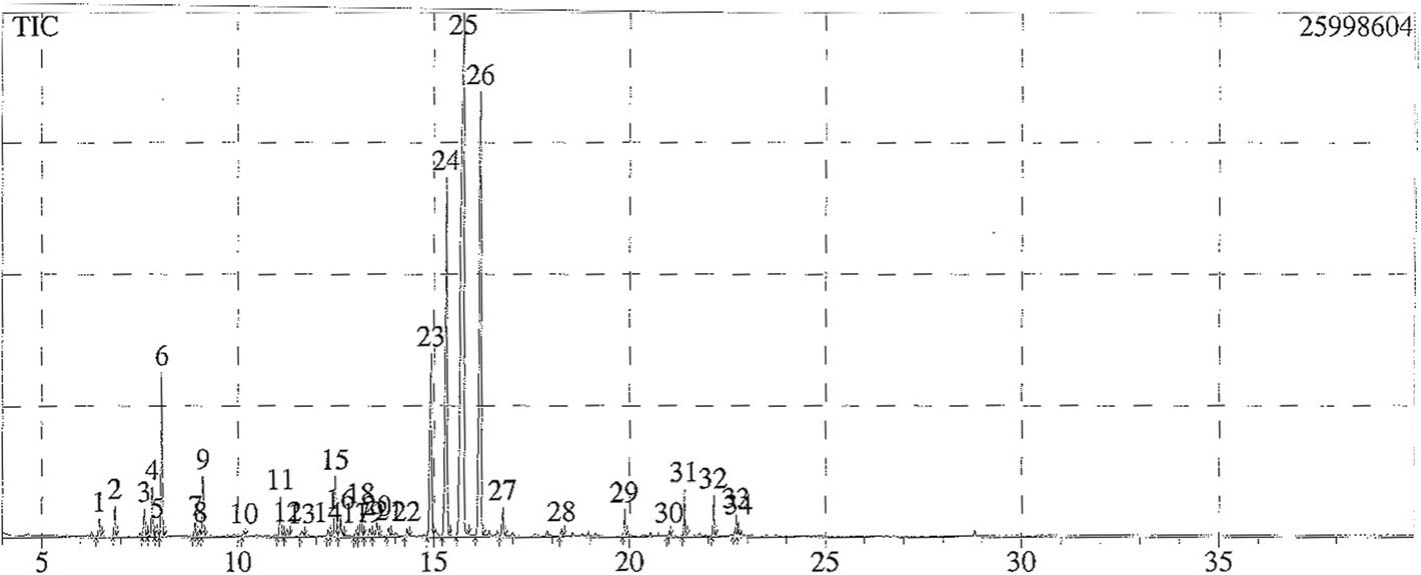
Representative GC/MS chromatogram of essential oil from *T. bovei*

The composition of the essential oil isolated from *T. bovei* is given in (Table 1 and Fig. 1). The essential oil was characterized as trans-geraniol (35.38 %), α-citral (20.37 %), β-citral (14.76 %), Cis-geraniol (7.38 %) and 3-octanol (4.38 %) as the major constituents.

**Table 1:** Chemical composition, concentrations (%) and calculated retention indices, of *T. bovei* essential oil as characterized by GC/MS analysis

Constituents	%	RI_C_	RI_L_
Trans-geraniol (Lemonol)	35.38	1261	1257
α-citral (Trans-citral)	20.37	1276	1278
β-citral (Cis-citral)	14.76	1246	1256
Cis-geraniol (nerol)	7.38	1233	1233
3-octanol	4.38	996	1012
DL-camphor	1.95	1148	1146
Eucalyptol(1,8) cineole	1.63	1032	1034
3-octanone	1.42	987	986
Thymol	1.28	1296	1292
β-linalool	1.17	1100	1098
β-farnesene	1.11	1456	1442
Geranylisobutyrate	1.02	1482	1450
L-borneol	0.9	1170	1149
Isocaryophyllene	0.89	1403	1397
Camphene	0.76	954	956
Bergamiol	0.76	1239	1242
Dihydrocarveol acetate	0.71	1302	1307
α-cyclocitral	0.66	1153	1124
β-ocimene	0.45	1056	1043
Geranyl propionate	0.36	1456	1453
β-myrcene	0.3	992	990
α-terpineol	0.26	1195	1188
α-limonene	0.22	1029	1027
Nerolidol	0.17	1495	1527
α-terpinene	0.16	1068	1053
α-phellandrene	0.1	996	1001
β-pinene	0.07	977	973
Total	98.62		
Yield (w/w) %	1.40 %		
Number of constituents	27		
Hydrocarbon monoterpenoid	2.06		
Oxygenated monoterpenoid	88.59		
Sesquiterpenoid hydrocarbon	2		
Oxygenated sesquiterpenoid	0.17		
Others	5.8		

*RI*_*C*_ calculated retention index, *RI*_*L*_ retention index obtained from literature

It can be seen that 88.59 % was oxygenated monoterpene, the high percentage of this class is due to the high percentage of trans-geraniol (lemonol) (35.38 %), α-citral (20.37 %), β-citral (14.76 %), cis-geraniol (7.38 %), DL-camphor (1.95 %), eucalyptol (1.63 %), thymol (1.28 %), β-linalool (1.17 %) and others comprising less than 1 % of the constituents.

Other major compounds belonging to monoterpene hydrocarbons were represented in camphene, β-ocimene, β-myrcene, α-terpinene, α-limonene, α-phellandrene and β-pinene and their total percentage was only 2.06 %. On the other hand, farnecene and isocaryophyllene were identified in a total percentage of 2 %, these belong to the hydrocarbon sesquiterpenoid group of essential oils. Oxygenated sesquiterpenoid was found only as nerolidol (0.17 %). Other compounds that are not terpenoid were identified such as 3-octanol (4.38 %) and 3-octanone (1.42 %).

### Anthelmintic activity

A perusal of Table 2 reveals that the *T. bovei* essential oil showed powerful anthelmintic activity which was even higher than the control drug piperazine citrate. In fact, at the same concentration of 10 mg/ml, the time of paralysis was 19.61 ± 0.88 and 24.25 ± 0.61 min for the essential oil and piperazine respectively. In addition, the time of death for the tested essential oil and control was 47.32 ± 0.94 and 62.96 ± 0.29 min respectively.

**Table 2:** Anthelmintic activity of *T. bovei* essential oil

Treatment	Concentration (mg/ml)	Time for paralysis (min) ± SD	Time for death (min) ± SD
Control	-	-	-
Piperazine citrate	10	24.25 ± 0.61**	62.96 ± 0.29 **
*T. bovei* essential oil	10	19.61 ± 0.88 ***	47.32 ± 0.94 ***
	40	11.32 ± 0.77 ***	33.51 ± 0.66**
	50	10.24 ± 0.27 **	29.95 ± 0.72 **
	75	7.96 ± 1.1 ***	22.59 ± 0.39 *
	100	3.38 ± 0.79 **	12.13 ± 0.79 ***

* *p* value <0.05, ** *p* value ≤ 0.001, and *** *p* value ≤ 0.0001

### Antioxidant activity

The free radical scavenging activity of the essential oil of *T. bovei* was tested by DPPH method using Trolox as a reference standard. The concentrations used ranged from 1–100 μg/ml for the essential oil as well as for Trolox. The results are shown in Table 3 that revealed that an excellent antioxidant activity was exhibited by *T. bovei* essential oil at IC_50_ ± SD of 3.41 ± 0.44 μg/ml, which was comparable to Trolox standard that showed IC_50_ ± SD of 3.25 ± 0.32 μg/ml, this means that the free radical scavenging activity for *T. bovei* essential oil was 95.30 % ± 1.3 of the activity of Trolox standard.

**Table 3:** Percentage inhibition of DPPH activity by *T. bovei* extract and Trolox

Concentration μg/ml	% inhibition by *T. bovei* essential oil ± SD	% of inhibition by Trolox ± SD
1	38.65 ± 1.08	40.6 ± 0.91
2	47.55 ± 1.34	48.7 ± 1.32
3	52.09 ± 1.05	56.09 ± 0.83
5	64.19 ± 1.32	60.12 ± 1.98
7	64.19 ± 1.83	80.12 ± 1.06
10	69.38 ± 1.33	87.95 ± 1.66
20	76.29 ± 2.12	88.71 ± 1.47
30	81.23 ± 1.43	91.55 ± 2.71
40	92.1 ± 1.65	91.56 ± 1.93
50	95.95 ± 1.40	99.45 ± 2.79
80	97.82 ± 1.87	99.55 ± 1.87
100	98.12 ± 1.58	99.55 ± 2.64

### Antibacterial and antifungal activities

The essential oil of *T. bovei* extracted by microwave-ultrasonic method used in this study exhibited potential bioactivity against the growth of all microbes examined in this study. With all of the studied pathogens, the highest antimicrobial activity (lowest MIC) against microbes examined was seen against *Pseudomonas aeruginosa, Staphylococcus aureus* and *Candida albicans* with MIC value of 0.25 mg/ml, while the MIC for *Escherichia coli* and MRSA was 0.5 mg/ml as shown in Table 4.

**Table 4:** Antimicrobial activities of *T. bovei* essential oil

Microorganism	MIC value (mg/ml) for *T. bovei* essential oil
*Escherichia coli* (ATCC 25922)	0.5
*Pseudomonas aeruginosa* (ATCC 27853)	0.25
*Staphylococcus aureus* (ATCC 25923)	0.25
*Staphylococcus aureus* (MRSA Positive)	0.5
*Candida albicans* (Clinical isolate)	0.25

## Discussion

Multi-antibiotic resistance is becoming one of the major global concerns. *Pseudomonas aeruginosa*, *Klebsiella pneumonia*, *Escherichia coli* and many other *ß-*lactamase inhibitors have become a major clinical problem. Increased attention has been focused on the usage of natural antimicrobial agents, especially from plant origins, due to their safety and efficacy as well as the fact that the majority of essential oils are classified as Generally Recognized As Safe (GRAS) [17].

In fact, natural products are used intensively as food preservatives, nutraceuticals as well as potential drugs for the treatment and prevention of various diseases and conditions including: cancer, cardiovascular disorders, aging and many others. For these reasons, in recent decades, worldwide studies have been conducted for the characterization, utilization and extraction of biological and pharmacological active compounds from plant origins [18].

The results of our phytochemical analysis showed consistent differences in the content of Palestinian *T. bovei* when compared with the only study which was conducted on the similar species by Tepe et al. 2011. Trans-geraniol, α-citral and β-citral were identified as major components of the Palestinian *T. bovei* essential oil while the major components of the Turkish *T. bovei* essential oil were carvacrol, p-cymene and thymol. This may be due to the differences between the two geographic and topographic areas, which may greatly affect their constituents [19, 20].

Moreover, these differences in the chemical composition may explain the differences in the antimicrobial activities. In fact, our *T. bovei* showed higher activity than the corresponding Turkish species, which were 0.5 and 31.25 for *Escherichia coli*, and 0.25 and 31.25 for *Candida albicans* respectively, which means that our studied species had more powerful antibacterial activity against studied microbial species than the Turkish *Thymus* species [20].

In addition, our results are in agreement with the literature with regards to the antimicrobial effects of these compounds which were reported in a study conducted by Pattnaik et al. 1996. In this study citral, linalool, geraniol, menthol and cineole were tested against eighteen bacterial strains and twelve fungal strains. The results showed that geraniol and citral are the active constituents in the aromatic plants with strong antibacterial and antifungal potentials [21].

Another study performed by Singh et al. 2012, found that geraniol and terpineol, the major components of geranium oil, were active against Gram-positive and Gram-negative pathogenic bacteria. Antifungal activity was also observed against yeast, *Dermatophytes* and *Aspergillus* species [22].

In addition, citral isomers such as trans-citral and cis-citral and geraniol were found to have potential antioxidant activity compared to Trolox standard, in studies performed by Choi et al. 2000 and Francesco et al., 2016 [23, 24].

In our study the free radical scavenging activity in comparison with Trolox standard for *T. bovei* essential oil was higher than *T. bovei* from Turkish origins which may again be caused by differences in the chemical compositions between the two species [20].

In fact, geraniol is an alcoholic monoterpenoid and is commercially used in flavors and fragrances. In addition to its aromatic odor, geraniol is known to exhibit insecticidal and repellent properties and is used as a natural pest control agent exhibiting low toxicity [25].

Accordingly, conducting research studies in order to discover other therapeutic agents is a striving challenge. Again, herbals are currently considered the most important door to be addressed. *Thymus bovei*, is extensively used as anthelmintic herb in the Palestinian heritage and folk medicine. Accordingly, it was extremely important to investigate the efficacy of its essential oil as an anthelmintic agent by comparing it with piperazine citrate.

The anthelmintic activity of *T. bovei* was tested in comparison with piperazine citrate. The obtained results showed significant potency for *T. bovei* against worms, which is supposed to be due to the presence of high content of geraniol in this plant species. In addition, these results showed a concentration dependent effect, since the time of paralysis and death decreased as the concentration of geraniol in the extract increased. These finding are in accordance with the literature, since plants with high geraniol content exhibited anthelmintic activity [25].

Further pharmacological and toxicological examination of *T. bovei* essential oil are needed to explore its synergistic effects with first line antibiotics, especially against bacteria that have developed resistance, as well as to study its safety.

## Conclusions

Our study showed that the essential oil extracted from *T. bovei* was comprised of a mixture of several volatile bioactive components, especially the oxygenated monoterpenoids, which showed strong anthelmintic, antioxidant and antimicrobial activities. This natural essential oil can be used for manufacturing of food supplements, cosmetics and pharmaceuticals formulations due to its potential biological and pharmacological activities. However, further investigation should be carried out on the *T. bovei* activities against other pathogens as well as cancer, and more studies are required to investigate its anthelmintic activity.

## Abbreviations

ATCC: American type culture collection
DMSO: Dimethyl sulfoxide
DPPH: 2,2-diphenyl-1-picrylhydrazyl
GC-MS: Gas chromatography mass spectrometry
GRAS: Generally recognized as safe
IC_50_: Half-maximal inhibitory concentration
MIC: Minimal inhibitory concentration
